# Neural and behavioral markers of inhibitory control predict symptom improvement during internet-delivered cognitive behavioral therapy for depression

**DOI:** 10.1038/s41398-024-03020-9

**Published:** 2024-07-23

**Authors:** Michelle Thai, Elizabeth A. Olson, Stefanie Nickels, Daniel G. Dillon, Christian A. Webb, Boyu Ren, William D. S. Killgore, Scott L. Rauch, Isabelle M. Rosso, Diego A. Pizzagalli

**Affiliations:** 1 Center for Depression, Anxiety and Stress Research, McLean Hospital, Belmont, MA USA; 2 Department of Psychiatry, Harvard Medical School, Boston, MA USA; 3 Psychiatric Biostatistics Laboratory, McLean Hospital, Belmont, MA USA; 4 Department of Psychiatry, University of Arizona College of Medicine, Tucson, AZ USA; 5 McLean Imaging Center, McLean Hospital, Belmont, MA USA

**Keywords:** Human behaviour, Neuroscience, Predictive markers, Prognostic markers, Depression

## Abstract

Poor inhibitory control contributes to deficits in emotion regulation, which are often targeted by treatments for major depressive disorder (MDD), including cognitive behavioral therapy (CBT). Brain regions that contribute to inhibitory control and emotion regulation overlap; thus, inhibitory control might relate to response to CBT. In this study, we examined whether baseline inhibitory control and resting state functional connectivity (rsFC) within overlapping emotion regulation-inhibitory control regions predicted treatment response to internet-based CBT (iCBT). Participants with MDD were randomly assigned to iCBT (*N* = 30) or a monitored attention control (MAC) condition (*N* = 30). Elastic net regression was used to predict post-treatment Patient Health Questionnaire-9 (PHQ-9) scores from baseline variables, including demographic variables, PHQ-9 scores, Flanker effects (interference, sequential dependency, post-error slowing), and rsFC between the dorsal anterior cingulate cortex, bilateral anterior insula (AI), and right temporoparietal junction (TPJ). Essential prognostic predictor variables retained in the elastic net regression included treatment group, gender, Flanker interference response time (RT), right AI-TPJ rsFC, and left AI-right AI rsFC. Prescriptive predictor variables retained included interactions between treatment group and baseline PHQ-9 scores, age, gender, Flanker RT, sequential dependency effects on accuracy, post-error accuracy, right AI-TPJ rsFC, and left AI-right AI rsFC. Inhibitory control and rsFC within inhibitory control-emotion regulation regions predicted reduced symptom severity following iCBT, and these effects were stronger in the iCBT group than in the MAC group. These findings contribute to a growing literature indicating that stronger inhibitory control at baseline predicts better outcomes to psychotherapy, including iCBT.

## Introduction

Deficits in executive functioning (EF), a heterogenous construct that encompasses processes including working memory, cognitive flexibility, and inhibitory control, are a prominent, clinically important feature of major depressive disorder (MDD) [1]. EF deficits are identifiable by the first depressive episode [2], persist in remitted patients [2, 3], do not significantly improve with antidepressant treatment [4], and are more severe following multiple episodes [5], suggesting that they may be either a vulnerability marker or a persistent consequence of MDD [2]. EF deficits can interfere with successful emotion regulation. Emotion regulation, a central skill in psychotherapy, including cognitive behavioral therapy (CBT), is the process of managing emotions through the application of any of several different strategies (e.g., cognitive reappraisal) [6], and it is of interest for MDD because symptoms of depression, such as excessive sadness, hopelessness, and the loss of positive mood, are signs of poor emotion regulation.

Overall, the existing literature suggests that poor baseline EF may be a prognostic marker signaling increased risk of poor treatment response [7, 8]. Few studies have examined EF as a predictor of response to psychotherapy in particular, including CBT, although there is some converging evidence that poor EF also predicts worse outpatient treatment response with psychotherapy or psychotropic medication [9] (though see [10]). Given their prevalence and persistence, it is important to understand how EF deficits in MDD may contribute to relapse, remission, and response to treatment. Specific aspects of EF may be particularly vulnerable in MDD. Inhibitory control is particularly relevant to MDD because successful emotion regulation requires an individual to inhibit the processing of negative information and disengage from negative information [11]. Poor inhibitory control then sets the stage for persistent low mood, the hallmark of depression.

Inhibitory control in MDD has been frequently assessed using the Flanker task, which measures selective attention, a subtype of inhibitory control that involves top-down, voluntary maintenance of goals to suppress attention to other stimuli [12, 13]. In the arrow Flanker task, participants must indicate the direction of a central “target” arrow surrounded by flanking arrows that either face in the same (‘congruent’: >>>>> or <<<<<) or opposite directions (‘incongruent’: >><>> or <<><<). The premise is that incongruent trials require greater inhibitory control because of the need to suppress conflicting information from the flankers [14]. Although the Flanker task does not classically contain emotionally valenced stimuli, “cold” and “hot” cognitive processes are not independent [15], and the Flanker task probes processes associated with emotion regulation. Notably, better Flanker performance is associated with greater likelihood of choosing reappraisal, a key emotion regulation strategy emphasized in CBT, over distraction [16].

The behavioral Flanker literature in MDD is mixed and group differences in Flanker interference effects compared to healthy controls (HC) have usually not been found [17–19]. However, more nuanced measures may be more sensitive to MDD deficits [20]. Post-error behavioral adjustments and analysis of sequential dependencies are two such measures. Following a trial on which an error occurs, healthy individuals typically slow (increase) their response time (RT) (the Rabbitt effect) and may also increase their accuracy (the Laming effect) [21]. These post-error adjustments may reflect the implementation of cognitive control following an error, orienting to an unexpected event (i.e., the error), increased motor inhibition, reduced sensitivity to sensory-perceptual information, and/or an increased threshold for the amount of evidence that must be accumulated to make a decision [22–25]. Healthy individuals also tend to perform better (more accurate, faster RT) on an incongruent trial when the incongruent trial follows another incongruent trial (i-i) than when it follows a congruent trial (c-i), a phenomenon called the Gratton effect [26]. The Gratton effect is thought to occur because an incongruent trial triggers the anterior cingulate cortex (ACC) to increase top-down control and increase attention to the target [27, 28], or due to priming of the association between the stimulus and response on the first trial [21, 29]. Individuals with MDD symptoms do not show typical post-error adjustments, possibly due to elevated affective responses to mistakes [30, 31], and show weaker Gratton effects [32] (though see [20, 31, 33]). Consistent with the general association between better baseline inhibitory control and better treatment outcomes, more normative post-error performance at baseline has been associated with greater symptom improvement following partial hospital treatment for MDD [34] (though see [20] for a negative finding on post-error group differences).

In contrast to the mixed behavioral literature, there is stronger evidence that neural alterations in inhibitory control systems occur in MDD and predict treatment outcomes. Neuroimaging methods may be more sensitive to subtle inhibitory control deficits that are not evident in behavioral data due to compensatory processes [35]. Individuals with MDD tend to show hypoactivity of inhibitory control brain regions [36, 37]. When MDD participants perform similarly to healthy individuals on inhibitory control tasks, they tend to demonstrate hyperactivity in prefrontal regions [38, 39]. Accordingly, when individuals with depression and HC showed similar error rates, individuals with depression showed greater dorsal ACC (dACC) activation on incorrect versus correct incongruent NoGo trials on a Flanker Go/NoGo task compared to HC, which may represent compensatory efforts and/or hypersensitivity to negative feedback [40]. Moreover, a growing literature demonstrates that brain regions implicated in inhibitory control overlap with those involved in emotion regulation [41, 42]. Because affective responses and motivation contribute to inhibitory control deficits, dysfunction in emotion regulation brain regions may also contribute to inhibitory control deficits in MDD. In particular, regions involved in inhibitory control and emotion regulation include portions of the anterior temporo-parietal junction (TPJ), anterior insula (AI), dACC, and inferior and middle frontal gyri [41, 42]. The dACC and inferior and middle frontal gyri have commonly been implicated in inhibitory control and emotion regulation [36, 41]. Although the AI and TPJ are heterogeneous brain regions involved in a number of different brain networks and functions, they are hubs that facilitate the engagement of appropriate brain networks for specific tasks, a function central to cognitive control [43, 44].

These overlapping regions are of particular interest because CBT aims to improve inhibitory control over emotional thoughts and processes (e.g., through teaching emotion regulation strategies like cognitive reappraisal). Critically, these overlapping inhibitory control-emotion regulation regions also form a resting state network [41, 45]. Resting state functional connectivity (rsFC) reflects the intrinsic connections between regions. Functionally connected brain regions exhibit synchronized activity at rest and the brain at rest uses 20% of bodily energy [46]. Brain energy consumption changes minimally with a task (5% or less [47]) and rsFC minimizes the potential confound of task performance, like reaction time or number of errors, on brain metrics [48]. As such, rsFC is a promising measure to probe the role of inhibitory control-emotion regulation regions in depression and treatment response. Indeed, rsFC within this shared network is related to inhibitory control behavioral performance [49, 50]. Specifically, greater dACC-supplementary motor area rsFC was related to neural markers of hyperresponsivity to errors [49] and greater dACC-dorsolateral prefrontal cortex (dlPFC) and insula-dlPFC rsFC was associated with lower conflict adaption or Gratton effect [50]. While prior studies have identified ACC activity as a predictor of treatment response in MDD [51–53], fewer studies have examined functioning throughout the overlapping inhibitory control-emotion regulation network. In one prior study, baseline rsFC between the right insula and right middle temporal gyrus was a significant predictor of symptom improvement during behavioral activation treatment for depression [54], though there was no comparison condition or placebo treatment.

Given CBT’s emphasis on cognitive restructuring and emotion regulation, we hypothesized that baseline inhibitory control and/or functioning within inhibitory control networks might relate to response to CBT. One implementation of CBT of growing importance due to its scalability (i.e., ability to reach many individuals at minimal cost) and increasingly widespread use is internet-based CBT (iCBT), which addresses multiple barriers to treatment access [55]. Studies of iCBT have identified factors associated with better treatment response [55–57] but, to date, we are not aware of studies relating baseline cognitive performance, and especially inhibitory control, to iCBT treatment response. Indeed, the literature on inhibitory control predicting response to CBT or psychotherapy is limited. Existing studies generally lack control groups, which makes it difficult to differentiate effects related to natural recovery vs. treatment-specific effects.

In sum, we investigated putative predictors of treatment response in participants with MDD enrolled in a randomized controlled trial of iCBT vs. a monitored attention control (MAC) condition. The set of predictors included behavioral measures of Flanker task performance, including the Flanker interference effect on accuracy and RT plus measures of post-error performance (Rabbitt-Laming effects) and sequential dependency (Gratton effects), together with rsFC between inhibitory control-emotion regulation network regions [42]. We hypothesized better Flanker performance and stronger rsFC between inhibitory control-emotion regulation network regions at baseline would predict better treatment response, and these effects would be stronger in the iCBT vs. MAC condition.

## Methods and materials

### Participants

Informed consent was obtained from two hundred sixty-six (266) participants to participate in the study. This study (ClinicalTrials.gov Identifier: NCT01598922) was approved by the Institutional Review Boards of McLean Hospital and Partners Healthcare and was conducted in accordance with the Declaration of Helsinki. The study entailed an initial screening visit (baseline assessment of symptoms, neuroimaging, and cognition), a 10-week iCBT treatment protocol, and a second in-person assessment. Inclusion criteria included primary diagnosis of current MDD and mild to moderate/severe self-reported depression scores on the Patient Health Questionnaire-9 (PHQ-9) [58] between 10 and 23. No participants were taking psychotropic medications. Participants who did not complete the treatment (iCBT: *n* = 7; MAC: *n* = 10) and one participant who inconsistently reported psychiatric history were excluded. In total, data from 60 participants were included (iCBT: *n* = 30; MAC: *n* = 30). See supplement for full inclusion/exclusion criteria, consort diagram, and comparisons between those who prematurely discontinued treatment and those who completed treatment.

### Treatment and self-report questionnaire (PHQ-9)

The goal of the overarching treatment study was to investigate whether iCBT would be more effective than MAC at improving depression symptoms, reducing negative cognitive biases, and normalizing brain functioning. This manuscript reports analyses of secondary outcomes to explore predictors of iCBT response. The original report of the treatment study described primary outcomes, and secondary outcomes related to volumetric findings were previously described; for full description of clinical procedures and outcomes, including attrition and the original clinical trial’s CONSORT diagram, please refer to these prior reports [57, 59]. Briefly, participants were randomly assigned to MAC or iCBT. Participants completed six online ‘lessons’ over a ten-week study period. At the start of each lesson, all participants completed symptom questionnaires, including the PHQ-9 [58]; for the MAC group, this was the full extent of their lessons. Participants in iCBT then completed six online CBT lessons along with weekly homework assignments.

The PHQ-9 is a self-report questionnaire assessing the 9 DSM-IV criteria for MDD (see supplement for more details) [58]. Although the Hamilton Rating Scale for Depression (HAMD) was also collected and was the primary outcome measure of this study’s original report [59], we focus on the PHQ-9 to increase the relevance of results to real-world implementation of online psychotherapy, which is more likely to utilize self-report as opposed to clinician rating scales. Because the HAMD is a gold-standard depression assessment, we also examined the HAMD as an outcome variable; please see the Results section and supplement for more details.

### Flanker task

Participants completed a modified Eriksen Flanker task outside of the scanner. Participants completed 30 practice trials and then five blocks, each consisting of 70 trials (46 congruent, 24 incongruent). See the supplement and Supplementary Table S1 for further details regarding the task and formulas for the output variables. Higher Flanker interference scores indicate more interference on incongruent trials (i.e., worse inhibitory control). Higher Gratton scores reflect greater ability to sustain selective attention across consecutive incongruent trials (better inhibitory control). Higher post-error adjustment scores reflect greater post-error adjustments (better inhibitory control).

### Resting state fMRI

A Siemens Tim Trio scanner (3.0 Tesla: Siemens, Erlangen, Germany) was used with a 32-channel head coil. Preprocessing was performed using SPM8 (update revision number 4667: http://www.fil.ion.ucl.ac.uk/spm/software/spm8/). Denoising and subsequent analytic steps were performed in the CONN toolbox (version 15.d: https://www.nitrc.org/projects/conn/) [60]. Further details of MRI image acquisition and resting state processing are presented in the supplement.

**Fig. 1:**
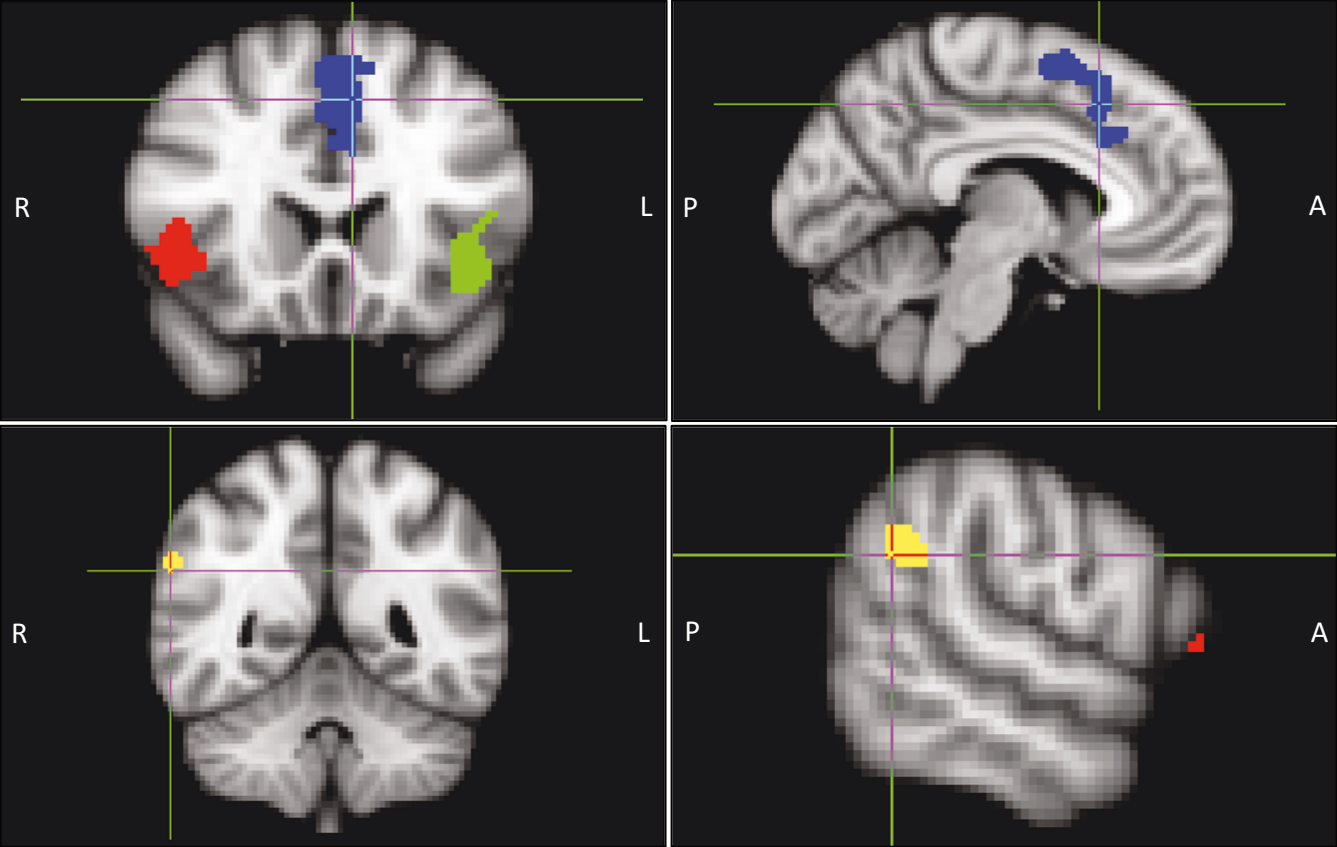
Locations of regions of interest (ROIs). Note. Blue: dorsal anterior cingulate cortex (dACC). Red: right anterior insula (AI). Green: left AI. Yellow: right temporoparietal junction (TPJ). ROIs were derived from Langner et al. [42], (Towards a human self-regulation system: Common and distinct neural signatures of emotional and behavioural control: http://anima.fz-juelich.de/studies/Langner_2018). The conjunction map for meta-analyses of cognitive emotion regulation (CER) and cognitive action regulation (CAR) was downloaded (CER_and_CAR_cFWE05.nii.gz, from http://anima.fz-juelich.de/studies/Langner_2018).

Regions of interest (ROIs) were derived from a meta-analysis by Langner et al. [42]. The conjunction map for meta-analyses of cognitive emotion regulation (CER) and cognitive action regulation (CAR) was downloaded (CER_and_CAR_cFWE05.nii.gz, from http://anima.fz-juelich.de/studies/Langner_2018). The map was subdivided into four contiguous clusters: right AI, left AI, frontal medial, and right TPJ (Fig. 1) and binarized. Pearson correlations between the time courses of the four ROIs were computed, and Fisher’s z-transformed values were extracted for further analysis. These regions do not overlap with those in our prior report of rostral ACC morphometry as a predictor of iCBT treatment response [57].

### Statistics

We imputed missing baseline data using a random forest based algorithm (missForest) [61, 62]. Missingness ranged from 0% (age, treatment group, gender, race, baseline PHQ-9, post-treatment PHQ-9) to 15% (rsFC). We also imputed data for Flanker variables that failed quality control (QC) metrics (7 Gratton and 12 post-error QC failures). We conducted chi-squared and *t*-tests to compare the iCBT to MAC groups on demographic variables (age, gender), baseline and post-treatment PHQ-9 scores, Flanker performance, and rsFC. Numeric variables were standardized using R’s scale function. We implemented elastic net regression (glmnet) [63] in R to predict post-treatment PHQ-9 scores using baseline variables. An elastic net regression selects variables to retain in the model while applying two penalties to avoid overfitting. We used 10-fold cross-validation (i.e., we split the data into ten subsets and each subset was used as a testing dataset while the remaining data was used as the training dataset) to select weights for the L1 and L2 penalties ($\alpha$) as well as the overall strength of the penalty ($\lambda$). In each model, entered variables included independent variables (described below) and the interactions between treatment group (iCBT vs MAC) and all independent variables. We implemented a three-stage approach to build the predictive model to determine which sets of variables are most predictive of post-treatment depression scores. In Stage 1, we conducted elastic net regressions with demographic/treatment variables (age, gender, treatment group, and baseline PHQ-9 score) and Flanker performance variables. In Stage 2, we replaced the Flanker variables in Stage 1 with emotion regulation-inhibitory control network rsFC variables. Variables retained in Stages 1 and 2 were entered in Stage 3, examining both Flanker and rsFC variables. In each stage, to account for the variability of variable selection, we repeated the elastic net regression analysis (which includes both the selection of $\alpha$ and $\lambda$ and the estimation of regression coefficients) 10,000 times and calculated the proportion of replicates in which a covariate had a non-zero regression coefficient. A variable was retained if the proportion was equal to or greater than 0.75 (i.e., had a non-zero regression coefficient in at least 7500 replicates). Because we are using the elastic net regression to build a prediction model, we report the proportion of replicates with non-zero coefficients; we do not report *p*-values. We include the confidence intervals and *p*-values in the supplement; of note, the testing results, however, are not accurate because the testing and training datasets overlap so although these *p*-values are all greater than 0.05, these variables still contribute to the prediction accuracy. In interpreting our results, we focus on variables with absolute value coefficients that exceed the average absolute value coefficient of all retained variables at each stage; variables below this threshold were considered non-essential predictors. See Supplementary Table S2 for results of the non-essential predictors. To evaluate the prediction performance of the three models, we additionally performed 10-fold cross-validation (on top of the 10-fold cross-validation for hyper-parameter tuning) during each of the 10,000 runs of the elastic net. During each run, we calculated the mean squared error (MSE) of the prediction for the fold for the three models, as well as the MSE for the prediction of a null model without any variables as predictors (i.e., by simply calculating the MSE for the test set minus the mean of the training set). We calculated the mean MSE of the elastic net regressions for the three stages and the null model over the 10,000 runs. We compared the mean MSEs of the different models to the null model and to the other elastic net models using corrected *t*-tests [64]. Finally, we fit an additional ordinary least squares linear regression model to the data with the essential predictors retained in Stage 3 to understand the associations of these variables to post-treatment PHQ-9 scores. Partial regression plots were generated to illustrate the adjusted effects of each retained variable using the ‘effects’ package in R; confidence bands are +/− the standard error of the fit.

## Results

### Demographics and descriptive statistics

The MAC group had lower baseline right AI-left AI rsFC (*t*(58) = 2.50, *p* = 0.015) and, as previously reported [59], higher post-treatment PHQ-9 scores (*t*(58) = 2.77, *p* = 0.007) than the iCBT group. There were no other significant group differences. See Table 1.

**Table 1:** Demographic information and descriptive statistics.

	MAC group (*n* = 30)				iCBT group (*n* = 30)				
	Mean (SD)	Skew	Kurtosis	SE	Mean (SD)	Skew	Kurtosis	SE	
Age	29.31 (7.01)	0.50	−0.89	1.28	29.86 (7.86)	0.76	−0.47	1.44	*t* = −0.29, *p* = 0.78
Gender (F/M)	(21/9)				(17/13)				X^2^ = 0.65, *p* = 0.42
Baseline PHQ-9	14.63 (3.55)	0.32	−0.39	0.65	13.33 (3.69)	−0.14	−0.80	0.67	*t* = 1.39, *p* = 0.17
Post Treatment PHQ-9	10.63 (4.60)	−0.08	−0.23	0.84	7.13 (5.16)	0.62	−0.49	0.94	*t* = 2.77, *p* = 0.01*
Flanker accuracy	0.22 (0.12)	0.47	−0.85	0.02	0.20 (0.14)	0.60	−0.85	0.02	*t* = 0.77, *p* = 0.44
Flanker RT	89.84 (15.45)	0.68	−0.38	2.82	86.14 (19.32)	0.91	0.92	3.53	*t* = 0.82, *p* = 0.42
Gratton accuracy	0.06 (0.12)	−0.50	−0.20	0.02	0.08 (0.06)	0.00	−0.77	0.01	*t* = −0.62, *p* = 0.54
Gratton RT	−3.71 (21.52)	−0.32	1.49	3.93	0.88 (17.07)	−0.63	0.88	3.12	*t* = −0.92, *p* = 0.36
Post-error accuracy	0.00 (0.04)	−0.14	0.23	0.01	-0.01 (0.03)	−0.54	0.15	0.01	*t* = 0.61, *p* = 0.55
Post-error RT	7.90 (23.46)	−0.09	0.09	4.28	6.79 (16.55)	−0.58	0.33	3.02	*t* = 0.21, *p* = 0.83
dACC-left AI rsFC	0.64 (0.24)	−0.47	−0.55	0.04	0.67 (0.18)	0.27	−1.09	0.03	*t* = −0.41, *p* = 0.68
dACC-right AI rsFC	0.53 (0.20)	−0.26	−0.25	0.04	0.57 (0.21)	−0.33	−0.27	0.04	*t* = −0.83, *p* = 0.41
dACC-TPJ rsFC	0.11 (0.20)	0.20	−0.13	0.04	0.14 (0.20)	0.80	1.31	0.04	*t* = −0.51, *p* = 0.61
Right AI-left AI rsFC	0.64 (0.25)	0.20	−0.24	0.05	0.79 (0.21)	−0.61	0.26	0.04	*t* = −2.50, *p* = 0.02*
Left AI-TPJ rsFC	0.17 (0.20)	0.09	−0.51	0.04	0.19 (0.20)	0.70	0.20	0.04	*t* = −0.29, *p* = 0.77
Right AI-TPJ rsFC	0.39 (0.23)	0.01	−0.79	0.04	0.38 (0.15)	−0.65	0.58	0.03	*t* = 0.15, *p* = 0.88

*MAC* monitored attention control, *iCBT* internet-based cognitive behavioral therapy, *SD* standard deviation, *SE* standard error, *F* female, *M* male, *PHQ-9* patient health questionnaire-9, *RT* response time, *dACC* dorsal anterior cingulate cortex, *AI* anterior insula, *rsFC* resting state functional connectivity, *TPJ* temporoparietal junction.

For all *t*-tests, the degree of freedom is 58; for the Chi-squared test, the degree of freedom is 1. For participant race and ethnicity: across the combined sample, participants reported that they were white (63.3%), Asian (13.3%), Black (5%), more than one race (5%), other (3.3%), unknown (10%); 10% reported Hispanic ethnicity, 31.7% did not provide ethnicity information, and 43.3% reported non-Hispanic ethnicity.

### Flanker behavioral results

Across both groups, the Flanker interference effects were significant for accuracy (*t*(59) = 12.84, *p* < 0.001, *d* = 1.66) and RT, *t*(59) = 39.08, *p* < 0.001, *d* = 5.04. The Gratton effect was significant for accuracy (*t*(59) = 5.80, *p* < 0.001, *d* = 0.75) but not RT, *t*(59) = −0.57, *p* = 0.57, *d* = 0.07. The post-error effect was significant for RT (*t*(59) = 2.82, *p* = 0.006, *d* = 0.36) but not for accuracy, *t*(59) = −1.16, *p* = 0.25, *d* = 0.15.

### Elastic net regressions predicting post-treatment PHQ-9 scores

#### Stage 1: Inhibitory control performance as predictors of symptom change

We entered demographic and treatment variables (age, gender, treatment group, and baseline PHQ-9 score) into the model, along with Flanker accuracy, Flanker RT, Gratton accuracy, Gratton RT, post-error accuracy, and post-error RT. Essential retained variables included treatment group; gender; and the interactions between treatment group and: gender, Flanker RT, Gratton accuracy, and post-error accuracy (Table 2; see Supplementary Table S2 for a complete list of retained variables).

**Table 2:** Variables retained in elastic net regressions predicting post-treatment PHQ-9 scores.

Variable	Proportion of replicates with non-zero coefficients
**Stage 1**	
Tx group	1.0000
Gender	0.9774
Tx group * Gender	0.9829
Tx group * Flanker RT	1.0000
Tx group * Gratton accuracy	1.0000
Tx group * Post-error accuracy	1.0000
**Stage 2**	
Tx group	1.0000
Right AI-TPJ rsFC	1.0000
Tx group * Age	1.0000
Tx group * Gender	0.9988
Tx group * Right AI-TPJ rsFC	1.0000
**Stage 3**	
Tx group	1.0000
Gender	0.9987
Flanker RT	1.0000
Right AI-TPJ rsFC	1.0000
Right AI-Left AI rsFC	1.0000
Tx group * Baseline PHQ-9	0.9999
Tx group * Age	1.0000
Tx group * Gender	1.0000
Tx group * Flanker RT	1.0000
Tx group * Gratton accuracy	1.0000
Tx group * Post-error accuracy	1.0000
Tx group * Right AI-TPJ rsFC	1.0000
Tx group * Right AI-Left AI rsFC	1.0000

For interpretation, only variables with absolute value coefficients that exceed the average absolute value coefficient of all retained variables at each step are included in this table. See supplementary Table S2 for a complete list of retained variables and proportion of replicates with non-zero coefficients.

*Tx* treatment, *PHQ-9* patient health questionnaire-9, *RT* response time, *AI* anterior insula, *rsFC* resting state functional connectivity, *TPJ* temporoparietal junction.

#### Stage 2: rsFC within inhibitory control-emotion regulation network regions as predictors of symptom change

We entered demographic and treatment variables (age, gender, treatment group, and baseline PHQ-9 score) into the model, along with rsFC values among dACC, left AI, right AI, and TPJ ROIs. Essential retained variables included treatment group; right AI-TPJ rsFC; and the interactions between treatment group and: age, gender, and right AI-TPJ rsFC (Table 2).

#### Stage 3: Inhibitory control performance and rsFC in inhibitory control-emotion regulation network regions as predictors of symptom change

We entered baseline PHQ-9 scores into the model, along with variables retained at Stage 1 and Stage 2, including age, treatment group, gender, Flanker accuracy and RT, Gratton accuracy and RT, post-error accuracy and RT, dACC-TPJ rsFC, dACC-left AI rsFC, right AI–TPJ rsFC, and left AI–right AI rsFC. Essential retained variables included treatment group; gender; Flanker RT; right AI-TPJ rsFC; left AI-right AI rsFC; and the interactions between treatment group and: baseline PHQ-9, age, gender, Flanker RT, Gratton accuracy, post-error accuracy, right AI–TPJ rsFC, and left AI-right AI rsFC (Table 2).

#### Model comparisons

The Stage 3 model (MSE_Stage3_ = 0.89) had a significantly lower MSE than the null (MSE_Null _= 0.99; M_diff_ = −0.098, SD_diff_ = 0.31, *t* = −3.14, *p* = 0.0017), Stage 1 (MSE_Stage1_ = 0.94; M_diff_ = −0.042, SD_diff_ = 0.21, *t* = −2.73, *p* = 0.0062), and Stage 2 models (MSE_Stage2_ = 0.99; M_diff_ = −0.092, SD_diff_ = 0.28, *t* = 3.43, *p* = 0.00059). The MSE values of the Stage 1 (*p* = 0.071) and Stage 2 (*p* = 0.91) models did not significantly differ from the null model.

### Linear model and directionality of effects for variables retained in the Stage 3 model

**Fig. 2:**
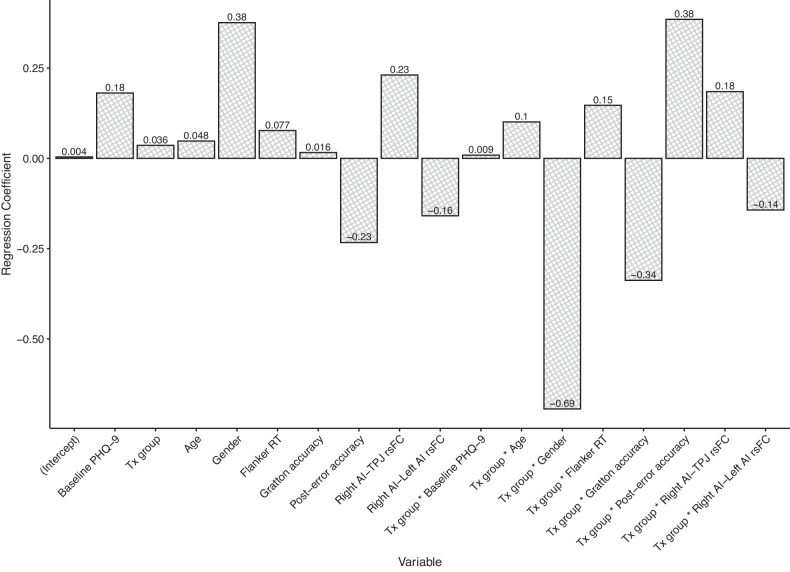
Plot of linear regression coefficients for model predicting post-treatment PHQ-9 scores, including essential variables retained in the elastic net regression (Stage 3). Note. Tx treatment, PHQ-9 patient health questionnaire-9, RT response time, AI anterior insula, rsFC resting state functional connectivity, TPJ temporoparietal junction.

We conducted a non-penalized linear regression using the variables retained in the Stage 3 model to obtain non-penalized coefficients estimates (see Fig. 2); overall, the model explained 46% of the variance in post-treatment PHQ-9 scores (R^2 ^= 0.46), F(17, 42) = 2.07, *p* = 0.03.

**Fig. 3:**
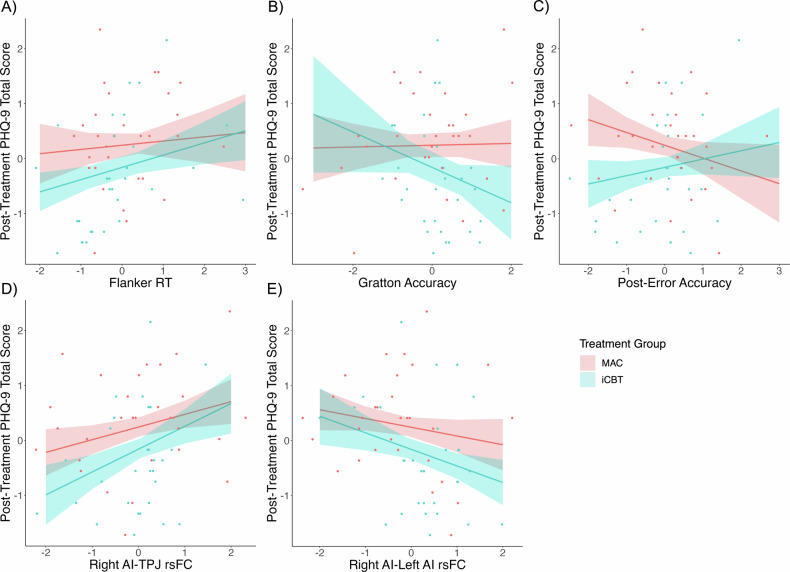
Partial regressions predicting post-treatment depression by treatment group by Flanker RT, Gratton accuracy, post-error accuracy, right AI-TPJ rsFC, and right AI-left AI rsFC. Note. Partial regressions predicting post-treatment depression by (**A**) Flanker RT by treatment group interaction, (**B**) Gratton accuracy by treatment group interaction, (**C**) post-error accuracy by treatment group interaction, (**D**) right AI-TPJ rsFC by treatment group interaction, and (**E**) right AI-left AI rsFC by treatment group interaction. MAC monitored attention control, iCBT internet-based cognitive behavioral therapy, PHQ-9 patient health questionnaire-9, RT response time, AI anterior insula, TPJ temporoparietal junction, rsFC resting state functional connectivity.

Higher Flanker RT (worse inhibitory control) was associated with higher post-treatment depression severity. This was qualified by an interaction with treatment group; the effect was in the same direction for both groups but stronger in iCBT than MAC (Fig. 3a).

Higher post-treatment depression severity also was associated with weaker right AI-left AI rsFC and stronger right AI-TPJ rsFC. Again, the effects were in the same direction in both groups but stronger in iCBT than MAC (Fig. 3d, e).

In addition, treatment group interacted with post-error and congruency sequence effects. Higher post-treatment depression severity was associated with lower Gratton accuracy (worse inhibitory control; Fig. 3b) but higher post-error accuracy (Fig. 3c) in the iCBT group only.

### Supplemental analyses

In the Supplement, we report first-order Pearson correlations between Baseline PHQ-9, Flanker, and rsFC variables and summarize several control analyses: (1) results with all variables entered in a single step, which were essentially identical to the stagewise analysis, (2) Stage 3 results controlling for a more comprehensive set of clinical variables, which were essentially identical to the stagewise analysis; (3) results in the restricted sample (*N* = 37) of individuals without missing data, which, as expected in this smaller, biased sample, were slightly different, but confirmed retention of Flanker variables as essential predictors; (4) results with baseline PHQ-9 scores as the outcome measure, which retained Flanker variables as essential predictors; (5) results with the HAMD as the outcome measure, which were similar but not identical given different clinical correlates of PHQ-9 and HAMD and confirmed retention of Flanker RT as an essential predictor; and (6) results with response status (defined as a ≥ 50% reduction in PHQ-9 scores) as the outcome measure, which, as expected due to the lower variance of the binary response status variable, were similar but not identical.

## Discussion

In summary, a set of variables related to inhibitory control at baseline predicted treatment outcomes in participants with MDD in a randomized controlled trial of iCBT. Weaker Flanker RT interference effects were associated with greater improvement in depression, and these effects were stronger in the iCBT than MAC group. In the iCBT but not MAC group, treatment outcomes were positively predicted by the Gratton accuracy effect but, surprisingly, negatively predicted by post-error accuracy. Additionally, lower right AI-TPJ rsFC and higher right AI-left AI rsFC were associated with greater improvements in depression, and these effects were stronger in the iCBT than MAC group.

Although participants in the current study showed some evidence of behavioral inhibitory control abnormalities at baseline (e.g., no Gratton effect on RT), they also showed some normative patterns, including the Gratton effect for accuracy and Rabbitt effect. The lack of a healthy control group prevents us from discerning whether these patterns were normative. Better baseline inhibitory control was associated with lower depression severity after treatment. This is consistent with prior literature suggesting that better baseline EF is associated with more favorable treatment outcomes in MDD [9, 65, 66]. To our knowledge, this study is the first to assess EF as a possible prescriptive (i.e., treatment-specific) indicator in a psychotherapy trial for MDD with a robust control condition. Better inhibitory control at baseline was associated with improved response to iCBT specifically (as opposed to non-specific factors shared with the control condition, such as interaction with study staff, and both groups had the same frequency and duration of interaction with study staff). While the Flanker RT effect was present in both groups, it was stronger in the iCBT than MAC group. Similarly, stronger sequential dependency effects on accuracy (better inhibitory control) predicted greater reduction in depression in the iCBT group only. In contrast, lower (worse inhibitory control) post-error accuracy adjustments were associated with *better* iCBT treatment outcomes. We previously found that depressed individuals did not show normal post-error accuracy improvements but instead showed lower accuracy following errors, which may have been related to abnormal affective/emotional responses to perceived mistakes [30, 31]. One possible interpretation of the unexpected direction of the post-error accuracy finding here is that iCBT might have been particularly effective for individuals with MDD who had this particular affective pattern at baseline (hypersensitivity to errors/mistakes). However, we did not explicitly assess hypersensitivity to errors to be able to evaluate this speculation.

Somewhat surprisingly, none of the rsFC variables that involved the dACC ROI were retained as essential predictors in the analysis. Instead, individual differences in rsFC involving the AI were related to differences in psychotherapeutic outcome. Specifically, better outcomes were associated with higher right AI-left AI rsFC and lower right AI-TPJ rsFC at baseline. The right AI is active during both successful and unsuccessful inhibition, and therefore may play a key role in detecting salient environmental features such as stop signals [67]. Salience network activation may ultimately signal a need for increased central executive control, deactivation of the default mode network (DMN), and/or a need for motor slowing [68, 69]. Indeed, the right AI has been associated with switching between the DMN and central executive brain networks [70, 71], and reduced anticorrelation between these networks is associated with treatment response [72, 73]. A recent meta-analysis identified the *left* AI as being involved in cognitive inhibition, while the bilateral AI was involved in inhibiting a prepotent motor response [13]. Stronger left AI-right AI rsFC at baseline may reflect a better capacity to coordinate across components of inhibitory control, including suppression of attentional distractors and/or suppression of motor responses elicited by those distractors. Left AI-right AI rsFC did not correlate with cognitive inhibition at baseline, however. Additionally, better outcomes were associated with lower rsFC between the right AI and TPJ. Deactivation of the TPJ increases inhibitory control, while activation of the TPJ increases reorienting [45]. Low right AI-TPJ rsFC might reflect a system ready to engage inhibitory control processes as opposed to stimulus-driven attentional reorienting. Indeed, at baseline, lower right AI-TPJ rsFC correlated with greater cognitive control (lower Flanker accuracy interference). The TPJ’s role in inhibitory control and orienting may explain why better clinical outcomes are associated with *higher* right AI-left AI but *lower* right AI-TPJ rsFC even though the AI and TPJ are within the same network. Finally, the AI also plays a critical role in error monitoring and adapting behavior following errors [74], which may explain its significance as a predictive indicator.

This study has several limitations. First, the iCBT group had (unexpectedly, given the random assignment) higher left AI-right AI rsFC at baseline than the MAC group. Second, strong inhibitory control might lead to better outcomes during iCBT, but other relationships between these variables also are possible. In particular, associations with additional factors such as emotion regulation might explain these findings. Third, replication is needed in a substantially larger sample, particularly for testing interactions. Fourth, it is unknown whether inhibitory control performance and rsFC within inhibitory control networks are associated with treatment response to iCBT beyond this study window (e.g., at 6-month or 12-month follow-ups). Fifth, additional limitations include the use of a self-report measure of symptom severity (see supplemental analyses using HAMD scores) and the fact that it is unknown whether these results would generalize to other treatment modalities. Finally, we focused exclusively on the Flanker and did not include other behavioral tasks. Although we chose the Flanker as it is a known behavioral probe of ACC functioning and the ACC is implicated in treatment response [38], the Flanker does not capture all aspects of inhibitory control, so these findings may not generalize to other measures of inhibitory control.

Together with other studies in the literature identifying predictors of response to different treatments for depression, these findings could potentially help to inform treatment selection for different patients to reduce the number of treatment trials patients must undergo before deriving benefit. Although lack of response to a depression treatment has major costs to the individual patient and society, including uncontrolled symptoms, time, effort, financial costs, and side effects, there is little guidance for personalized treatment selection. Choosing an effective initial treatment is critical given that patients who do not respond to an initial treatment are less likely to benefit from future treatments [75]. Notably, our findings align with other studies predicting treatment response from EF performance, suggesting that more preserved EF is generally associated with better treatment outcomes to antidepressant medications, transcranial direct current stimulation, CBT, and psychodynamic therapy [8]. Of note, ketamine, deep brain stimulation, cognitive rehabilitation, problem-solving therapy and supportive therapy have shown opposite effects with poorer executive functioning being associated with better treatment response, and no associations were found between EF and response to electroconvulsive therapy (ECT; see [8] for a review), suggesting that EF performance may have potential for treatment-specific prediction.

Recent reviews and meta-analyses have implicated rsFC of brain regions outside of our inhibitory control-emotion regulation network in predicting antidepressant treatment response, highlighting the importance of DMN rsFC and subgenual and rostral ACC rsFC in predicting treatment response across a variety of treatments, including transcranial magnetic stimulation (TMS), antidepressant medication, and psychotherapy [76, 77]. However, the majority of participants in this meta-analysis were treated with TMS and antidepressant medication and only 39 subjects participating in CBT were included [76]. Functioning within inhibitory control-emotion regulation regions and the prefrontal cortex (PFC) has been implicated as predictors of treatment response when examining brain functioning during response inhibition and as predictors of CBT response in particular. Greater insula and PFC activation during successful inhibition and dlPFC connectivity during response inhibition have been found to predict treatment response to antidepressant medications [78, 79]. Greater positive subcallosal cingulate-ventrolateral PFC/insula and subcallosal cingulate-ventromedial PFC rsFC was predictive of CBT treatment response (whereas the opposite pattern was found for antidepressant medication) [80]. Electrophysiological indices of intact response inhibition have also been predictive of antidepressant medication and CBT treatment response [81]. These findings along with the current results suggest strong functioning of and pathways among inhibitory control-emotion regulation brain regions may promote response to CBT specifically. Larger studies with comprehensive clinical and neurocognitive assessments randomizing individuals with depression to different treatment modalities are needed to generate and fully test predictive models that can determine which treatment a patient is most likely respond to.

In summary, we have identified a set of predictors of treatment response in unmedicated participants with MDD enrolled in a randomized controlled trial of iCBT, assigned to either iCBT or a control treatment. These predictors included behavioral measures of better inhibitory control as well as worse post-error behavioral adjustments. Additionally, rsFC variables were retained in the model, including connectivity across nodes involved in cognitive control and emotional regulation (particularly right AI-TPJ and right AI-left AI rsFC). Together, these findings contribute to a growing body of literature indicating that stronger inhibitory control at baseline predicts better outcomes during CBT.

### Supplementary information

Supplemental Material

## Data Availability

Data are available from the corresponding author upon request.
