# Supplementary material for: Neural and behavioral markers of inhibitory control predict symptom improvement during internet-delivered cognitive behavioral therapy for depression
Source: Transl Psychiatry. 2024 Jul 23;14:303. doi: 10.1038/s41398-024-03020-9 (PMC11266709; doi:10.1038/s41398-024-03020-9)
Supplement: Supplementary file 1 — Supplemental Material [file 41398_2024_3020_MOESM1_ESM.docx]

**Supplement**

**METHODS**

*Inclusion/exclusion criteria*:

Inclusion criteria included current DSM-IV MDD, mild to moderate/severe depression defined as Patient Health Questionnaire-9 (PHQ-9)^1^ score between 10 and 23, age 18 to 45, English fluency, regular access to a phone and computer, absence of psychotropic medications for at least the past 2 weeks (6 weeks for fluoxetine and 6 months for neuroleptics), and right-handedness. Exclusion criteria included suicidal ideation (initial PHQ-9 item 9 score 2 or greater), lifetime bipolar or schizophrenia spectrum disorder, lifetime substance abuse or dependence, current alcohol abuse or dependence, past alcohol dependence, past-year recreational drug use (except cannabis), past month cannabis use, current participation in CBT, history of electroconvulsive therapy, education less than 9 years, and MRI contraindications.

*Self-Report Questionnaire (PHQ-9)*:

The PHQ-9 is a self-report questionnaire assessing the 9 DSM-IV criteria for MDD. PHQ-9 items are scored from 0 to 3, summed to produce a total score between 0 and 27. Initial validation studies demonstrated that the PHQ-9 has adequate internal reliability (Cronbach’s alpha = 0.86-0.89), test-retest reliability (0.84), and concurrent validity based upon concordance with clinical interviews.^63^ Reduction in PHQ-9 scores in the iCBT group (6.2 point decrease) was comparable to that seen in other recent studies of iCBT for MDD.^64^

*Additional questionnaires used in supplemental analyses:*

Mood and Anxiety Symptom Questionnaire (MASQ, 62-item brief form). The MASQ is a self-report questionnaire designed to assess Clark & Watson’s tripartite model of anxiety and depression, which proposes a nonspecific/overarching negative affect factor, a somatic tension/hyperarousal factor specific to anxiety, and a low positive affect factor specific to depression.^2,3,3,4^ Participants provide past-week ratings of intensity of each item from 1 (“very slightly or not at all”) to 5 (“extremely”). Subscales used in this study included MASQ Anhedonic Depression (n = 22 items, related to loss of interest (8) and reverse-scored high positive affect (14)) and MASQ Anxious Arousal (n = 17 items) subscales.

Beck Anxiety Inventory (BAI).^5^ The BAI is a 21-item self-report questionnaire assessing symptoms of past-week anxiety. Participants rate how much they have been bothered by each symptom during the past week, from 0 (“not at all”) to 3 (“severely, I could barely stand it”), summed to produce a total score ranging from 0-63. Validation studies demonstrated that the BAI has adequate internal reliability (Cronbach’s alpha = 0.92) and adequate concurrent validity based upon concordance with other self-report measures of anxiety and psychological distress.^6–8^

*Flanker task:*

Participants completed a modified Eriksen Flanker task on a study laptop outside of the scanner. They first completed a practice session consisting of 30 trials (15 congruent, 15 incongruent). They were instructed to respond as quickly as possible by pressing the ‘c’ key with their left index finger when the center arrow pointed left (<<<<< or >><>>) or by pressing the ‘m’ key with their right index finger when the center arrow pointed right (>>>>>, <<><<). For each individual, the 85th percentile of their practice response time (RT) distribution was used as a deadline for responding during the first block of the actual task.

Prior to the actual task, participants were prompted, “It is very important that you respond as quickly and accurately as possible. To help you stay on track, you will receive negative feedback if you go too slowly. If you respond too slowly, the word “TOO SLOW” will appear on the screen. If you see these words, please try to speed up.” Participants completed five blocks, each consisting of 70 trials (46 congruent, 24 incongruent). The 85th percentile of RT in the prior block was used to establish a response deadline for blocks 2-5; the deadline for block 1 was set based on the practice trials. Each trial consisted of: flanking arrow presentation (100 ms), addition of the central arrow (50 ms), fixation cross (response period: 1400 ms), presentation of TOO SLOW feedback or extended fixation cross (300 ms), and variable duration final fixation cross (200-400 ms). The total trial duration varied from 2050-2250 ms. At the end of each block, participants were given feedback to ensure an acceptable speed-accuracy tradeoff: participants making too few errors on incongruent trials (2 or fewer errors per block) were instructed, “Remember to respond as QUICKLY as possible while still being accurate,” which participants making too many errors on incongruent trials (6 or more per block) were instructed, “Remember to respond as ACCURATELY as possible while still being fast.” If neither applied, the end-of-block screen read, “Please respond as quickly and accurately as possible.”

*Quality control*: Outlier RTs were defined as those that were implausibly short (< 150 ms), or those greater than 3 standard deviations from the participant’s mean RT (calculated separately for congruent and incongruent trials). These trials were excluded from further analysis. Participants were retained if their data set included: 1) no more than 35 RT outliers, 2) at least 200 congruent and 90 incongruent trials with non-outlier RTs, and 3) congruent and incongruent trial accuracies of at least 50%. For the post-error effects, an additional QC check was the need for at least 6 trials that followed an error on an incongruent trial.^9^

| Flanker interference accuracy | %Correct _Congruent trials_  ̶ %Correct _Incongruent trials_ |
| --- | --- |
| Flanker interference RT | Mean RT _Incongruent correct trials_  ̶ Mean RT _Congruent correct trials_ |
| Gratton accuracy | %Correct _Incongruent trials following correct incongruent trials_  ̶ %Correct _Incongruent trials following correct congruent trials_ |
| Gratton RT | Mean RT _Incongruent trials following correct congruent trials_  ̶ Mean RT _Incongruent trials following correct incongruent trials_ |
| Post-error accuracy | %Correct _Trials following incorrect incongruent trials_  ̶ %Correct _Trials following correct incongruent trials_ |
| Post-error RT | Mean RT _Trials following incorrect incongruent trials_  ̶ Mean RT _Trials following correct incongruent trials_ |

Supplementary Table S1. Flanker task performance summary measures

*Note.* RT = response time.

*Resting state fMRI:*

MR image acquisition: A Siemens Tim Trio scanner (3.0 Tesla: Siemens, Erlangen, Germany) was used with a 32-channel head coil. We collected structural T1-weighted 3D magnetization-prepared rapid gradient-echo (MPRAGE) images over 176 sagittal slices (TR / TE / flip angle = 2100ms/2.3ms/12°, 256 × 256 matrix; voxel size = 1.0 × 1.0 × 1.0 mm^3^). For resting state fMRI, 180 T2*-weighted echoplanar images (EPI) were collected over 34 transverse interleaved slices (TR / TE / flip angle = 2000ms/30ms/90°) with voxel size = 3.5 x 3.5 x 3.5 mm^3^, 224 mm field of view.

Image processing and analysis: Standard preprocessing was performed using SPM8 (update revision number 4667: http://www.fil.ion.ucl.ac.uk/spm/software/spm8/) running in MATLAB R2014a. Denoising and subsequent analytic steps were performed in the CONN toolbox (version 15.d: <https://www.nitrc.org/projects/conn/>).^65^

Participants received standardized instructions before completing the resting state scan (“For this scan, we want you to rest quietly with your eyes open and let your mind wander. Do not move and do not fall asleep. Just let your mind daydream for the next few minutes as the scanner operates. Do you have any questions?”).

Preprocessing steps included realignment and unwarping, slice timing correction, coregistration to the structural image, segmentation, spatial normalization in Montreal Neurological Institute (MNI) space, smoothing (full-width half maximum [FWHM] = 6mm), and reslicing at 2 x 2 x 2 mm.

For each participant, Artifact Detection Tools (ART, www.nitrc.org/projects/artifact_detect) was used to identify time points with excessive head motion (> 1 mm from the prior frame) or spikes in global signal intensity (> 3 SD from mean intensity across functional scans). For all subjects, the first volume was flagged as an outlier because of possible field inhomogeneity at the beginning of the scan. Participants with more than 15% flagged volumes were removed from the analysis. Outlier volumes were regressed out during first-level modeling. Additionally, parameters estimating head motion were regressed out at the first level (three translation parameters, three rotation parameters, and one composite parameter reflecting maximum scan-to-scan movement).

Correction for physiological noise (e.g., noise related to cardiac and respiratory activity) was performed in the CONN functional connectivity toolbox (version 15.d: <https://www.nitrc.org/projects/conn/>;^10^). Sources of noise were estimated using CompCor,^11^ which uses principal components analysis to estimate physiological noise from white matter and cerebrospinal fluid and remove it from the BOLD time series. Within CONN, the denoising step therefore included linear regression of the outlier and motion parameters, white matter, CSF, and the main effect of rest as well as its first temporal derivative (to eliminate ramping effects). Following the regression, a band-pass filter (0.008 to 0.09 Hz) was applied to the residual timeseries. The subsequent analysis was performed using the residual BOLD time course at each voxel.

**Supplementary Figure S1**

*Consort Diagram*

Eligible and completed diagnostic screening: n = 94

Allocated to MAC: n = 40

Allocated to iCBT: n = 37

Completed baseline measures and were randomized to treatment: n = 77

Completed post treatment measures:

n = 30

Completed post treatment measures: n=30

Outcome Measures

rsFC: n=51

Flanker:

Interference Accuracy/RT: n = 59

Gratton Accuracy/RT: n = 53

Post-Error Accuracy/RT: n = 48

*Note.* iCBT = internet-based cognitive behavioral therapy; MAC = monitored attention control; RT = response time; rsFC = resting state functional connectivity.

1 participant was missing Flanker task data, 7 participants failed Flanker and Gratton quality check, and 12 participants failed the Post-Error quality check (i.e., they had less than 7 trials).^9^ Participants with imaging data quality issues or more than 15% flagged volumes were removed from the rsfMRI analysis. There were no significant differences in terms of gender, race, and age between those who completed treatment and those who prematurely discontinued treatment, all p’s ≥ .10. PHQ-9 depression symptom severity was significantly higher in those who did not complete treatment (M = 17.59, SD = 3.99) compared to those who completed treatment (M = 13.98, SD = 3.65), t(24.13) = 3.35, p = .003.

**RESULTS**

Supplementary Table S2. All variables retained in elastic net regressions predicting post-treatment PHQ-9 scores

| **Variable** | **Proportion of replicates with non-zero coefficients** | **Average Coefficient** |
| --- | --- | --- |
| **Stage 1** | |  |
| Tx group | 1 | -0.203 |
| Baseline PHQ-9 | 1 | 0.066 |
| Age | 0.9784 | 0.033 |
| Gender | 0.9774 | 0.099 |
| Flanker RT | 1 | 0.079 |
| Gratton accuracy | 0.8703 | 0.018 |
| Gratton RT | 0.9722 | -0.032 |
| Post-error RT | 0.9931 | -0.047 |
| Tx group * Baseline PHQ-9 | 0.9983 | 0.082 |
| Tx group * Age | 1 | 0.082 |
| Tx group * Gender | 0.9829 | -0.133 |
| Tx group * Flanker accuracy | 0.9428 | -0.034 |
| Tx group * Flanker RT | 1 | 0.102 |
| Tx group * Gratton accuracy | 1 | -0.157 |
| Tx group * Post-error accuracy | 1 | 0.121 |
| Tx group * Post-error RT | 0.7533 | 0.018 |
| **Stage 2** | |  |
| Tx group | 1 | -0.23 |
| Baseline PHQ-9 | 1 | 0.103 |
| Age | 1 | 0.074 |
| Gender | 0.9845 | 0.084 |
| dACC-TPJ rsFC | 0.9987 | -0.03 |
| dACC-Left AI rsFC | 0.9959 | 0.055 |
| Right AI-TPJ rsFC | 1 | 0.124 |
| Right AI-Left AI rsFC | 1 | -0.084 |
| Tx group * Baseline PHQ-9 | 0.9164 | 0.057 |
| Tx group * Age | 1 | 0.16 |
| Tx group * Gender | 0.9988 | -0.178 |
| Tx group * dACC-TPJ rsFC | 0.998 | -0.079 |
| Tx group * Right AI-TPJ rsFC | 1 | 0.248 |
| Tx group * Right AI-Left AI rsFC | 1 | -0.11 |
| **Stage 3** | |  |
| Tx group | 1 | -0.156 |
| Baseline PHQ-9 | 1 | 0.059 |
| Age | 1 | 0.043 |
| Gender | 0.9987 | 0.07 |
| Flanker RT | 1 | 0.065 |
| Gratton accuracy | 0.9193 | 0.007 |
| Gratton RT | 0.9999 | -0.032 |
| Post-error RT | 1 | -0.041 |
| Right AI-TPJ rsFC | 1 | 0.072 |
| Right AI-Left AI rsFC | 1 | -0.066 |
| dACC-TPJ rsFC | 0.9926 | -0.018 |
| dACC-Left AI rsFC | 0.9927 | 0.028 |
| Baseline PHQ-9 * Tx group | 0.9999 | 0.066 |
| Tx group * Age | 1 | 0.077 |
| Tx group * Gender | 1 | -0.134 |
| Tx group (MAC) * Flanker accuracy | 0.9193 | 0.007 |
| Tx group * Flanker accuracy | 0.9765 | -0.031 |
| Flanker RT * Tx group | 1 | 0.076 |
| Tx group * Gratton accuracy | 1 | -0.128 |
| Tx group (MAC) * Post-error accuracy | 1 | -0.042 |
| Tx group * Post-error accuracy | 1 | 0.096 |
| Tx group * Post-error RT | 0.9194 | 0.015 |
| Tx group * dACC-TPJ rsFC | 0.9896 | -0.03 |
| Tx group * Right AI-TPJ rsFC | 1 | 0.124 |
| Tx group * Right AI-Left AI rsFC | 1 | -0.088 |

*Note.* Tx = treatment; PHQ-9 = Patient Health Questionnaire-9; RT = response time; AI = anterior insula; rsFC = resting state functional connectivity; TPJ = temporoparietal junction; dACC = dorsal anterior cingulate cortex.

|  | **Post-Treatment PHQ-9** | | |
| --- | --- | --- | --- |
| *Predictors* | *Estimates* | *CI* | *p* |
| Intercept | 0.004 | -0.65 – 0.66 | 0.991 |
| Baseline PHQ-9* | 0.18 | -0.19 – 0.55 | 0.326 |
| Tx group | 0.036 | -0.87 – 0.94 | 0.937 |
| Age* | 0.048 | -0.38 – 0.48 | 0.822 |
| Gender | 0.38 | -0.49 – 1.24 | 0.385 |
| Flanker RT | 0.077 | -0.41 – 0.56 | 0.751 |
| Gratton accuracy* | 0.016 | -0.38 – 0.42 | 0.934 |
| Post-error accuracy* | -0.23 | -0.69 – 0.22 | 0.310 |
| Right AI-TPJ rsFC | 0.23 | -0.15 – 0.61 | 0.225 |
| Right AI-Left AI rsFC | -0.16 | -0.55 – 0.23 | 0.415 |
| Tx group * Baseline PHQ-9 | 0.009 | -0.51 – 0.52 | 0.972 |
| Tx group * Age | 0.1 | -0.50 – 0.71 | 0.737 |
| Tx group * Gender | -0.69 | -1.87 – 0.49 | 0.242 |
| Tx group * Flanker RT | 0.15 | -0.44 – 0.73 | 0.615 |
| Tx group * Gratton accuracy | -0.34 | -1.13 – 0.46 | 0.394 |
| Tx group * Post-error accuracy | 0.38 | -0.23 – 1.00 | 0.215 |
| Tx group * Right AI-TPJ rsFC | 0.18 | -0.46 – 0.83 | 0.565 |
| Tx group * Right AI-Left AI rsFC | -0.14 | -0.73 – 0.44 | 0.626 |
| Observations | 60 | | |
| R^2^ / R^2^ adjusted | 0.456 / 0.236 | | |

Supplementary Table S3. Linear regression predicting post-treatment PHQ-9 scores, including essential variables retained in the elastic net regression (Stage 3).

*Note.* Tx = treatment; PHQ-9 = Patient Health Questionnaire-9; RT = response time; AI = anterior insula; rsFC = resting state functional connectivity; TPJ = temporoparietal junction. See also Figure 1. *included in linear model although it was not retained in the elastic net because it was part of a retained interaction.

Supplementary Table S4. *Descriptive statistics for additional questionnaires used in supplemental analyses:*

|  | MAC group (n = 30) | | | | iCBT group (n = 30) | | | |  |
| --- | --- | --- | --- | --- | --- | --- | --- | --- | --- |
|  | Mean (SD) | Skew | Kurtosis | SE | Mean  (SD) | Skew | Kurtosis | SE |  |
| MASQ Anxious Arousal | 24.10  (7.06) | 1.59 | 2.20 | 1.29 | 22.73  (5.29) | 1.61 | 2.46 | 0.97 | t = 0.85,  p = 0.40 |
| MASQ Anhedonic Depression | 86.57  (8.54) | -0.60 | -0.48 | 1.56 | 82.33  (7.64) | -0.08 | -0.90 | 1.40 | t = 2.02,  p = 0.048* |
| BAI | 11.91  (9.08) | 0.60 | -0.72 | 1.66 | 11.60  (6.88) | 0.35 | -1.10 | 1.26 | t = 0.15,  p = 0.88 |

*Note.* MAC = monitored attention control; iCBT = internet-based cognitive behavioral therapy; SD = standard deviation; SE = standard error; MASQ = Mood and Anxiety Symptom Questionnaire; BAI = Beck Anxiety Inventory.

For all t-tests, the df is 58.

Supplementary Table S5. *First-order Pearson correlations between Baseline Depression, Flanker variables, and rsFC variables (n = 60)*

|  | PHQ-9 | dACC-  left AI | dACC-  right AI | dACC-TPJ | Left AI-  right AI | Left AI-TPJ | Right AI-TPJ |
| --- | --- | --- | --- | --- | --- | --- | --- |
| PHQ-9 | 1.000 | -0.085 | -0.207 | 0.047 | -0.199 | 0.136 | -0.066 |
| Flanker RT | 0.054 | 0.192 | 0.021 | -0.154 | 0.133 | 0.182 | 0.211 |
| Flanker Accuracy | -0.010 | 0.137 | 0.104 | 0.018 | 0.199 | 0.235 | 0.260* |
| Gratton RT | -0.180 | 0.100 | 0.152 | 0.265* | -0.156 | 0.001 | -0.105 |
| Gratton Accuracy | 0.044 | 0.064 | -0.020 | -0.168 | 0.234 | -0.060 | 0.192 |
| Post-error RT | -0.235 | 0.035 | 0.054 | 0.067 | 0.048 | 0.078 | 0.175 |
| Post-error Accuracy | 0.197 | -0.007 | -0.024 | 0.256* | -0.090 | 0.178 | 0.233 |

*Note.* PHQ-9 = Patient Health Questionnaire-9; RT = response time; dACC = dorsal anterior cingulate cortex; AI = anterior insula; TPJ = temporoparietal junction.

*p < 0.05

*A. Entering all variables simultaneously into the elastic net as a single step*

As an alternative to the multi-stage process presented here, we explored entering all variables into the model in a single stage. Given the large number of entered variables, we used a lower cutoff (5000 out of 10,000 replicates) to consider a variable retained. We entered basic demographic/treatment variables, including age, gender, treatment group, and baseline PHQ-9 score into the model, along with Flanker performance variables including Flanker accuracy, Flanker RT, Gratton accuracy, Gratton RT, post-error accuracy, and post-error RT; and connectivity values among dACC, left AI, right AI, and TPJ ROIs (n = 32 variables, including interactions and the intercept). The retained variables that exceed the average absolute coefficient of all retained variables (.05) using this approach were largely similar to the essential predictors in Stage 3 in the main manuscript, suggesting that our results were not sensitive to the setup of the multi-stage approach. The only difference compared to the multi-stage approach in the main results was that baseline PHQ-9 emerged as an essential predictor in the single step approach. See Supplementary Table S6.

Supplementary Table S6

| **Variable** | **Proportion of replicates with non-zero coefficients** | **Average Coefficient** |
| --- | --- | --- |
| Tx group (MDD)☨ | 1 | -0.151 |
| Baseline PHQ-9☨ | 1 | 0.055 |
| Age | 0.9999 | 0.038 |
| Gender☨ | 0.9857 | 0.057 |
| Flanker accuracy | 0.7829 | -0.008 |
| Flanker RT☨ | 1 | 0.062 |
| Gratton accuracy | 0.7852 | 0.012 |
| Gratton RT | 0.9979 | -0.03 |
| Post-error accuracy | 0.7827 | 0.008 |
| Post-error RT | 0.9998 | -0.041 |
| Left AI-TPJ rsFC | 0.7827 | -0.004 |
| Right AI-TPJ rsFC☨ | 1 | 0.066 |
| Right AI-Left AI rsFC☨ | 1 | -0.058 |
| Dorsal ACC-TPJ rsFC | 0.9611 | -0.015 |
| Dorsal ACC-left AI rsFC | 0.9446 | 0.025 |
| Dorsal ACC-right AI rsFC | 0.7827 | -0.009 |
| Tx group (MDD) * Baseline PHQ-9☨ | 0.9999 | 0.06 |
| Tx group (MDD) * Age☨ | 1 | 0.071 |
| Tx group (MDD) * Gender☨ | 0.9999 | -0.121 |
| Tx group (MDD) * Flanker accuracy | 0.845 | -0.027 |
| Tx group (MDD) * Flanker RT☨ | 1 | 0.072 |
| Tx group (MDD) * Gratton accuracy☨ | 1 | -0.123 |
| Tx group (MDD) * Gratton RT | 0.7827 | -0.015 |
| Tx group (MDD) * Post-error accuracy☨ | 1 | 0.088 |
| Tx group (MDD) * Post-error RT | 0.7827 | 0.012 |
| Tx group (MDD) * Left AI-TPJ rsFC | 0.7827 | 0.017 |
| Tx group (MDD) * Right AI-TPJ rsFC☨ | 1 | 0.113 |
| Tx group (MDD) * Right AI-Left AI rsFC☨ | 1 | -0.083 |
| Tx group (MDD) * Dorsal ACC-TPJ rsFC | 0.8943 | -0.023 |
| Tx group (MDD) * Dorsal ACC-left AI rsFC | 0.783 | -0.019 |
| Tx group (MDD) * Dorsal ACC-right AI rsFC | 0.9329 | -0.016 |

*Note.* Tx = treatment; MDD = Major Depressive Disorder; PHQ-9 = Patient Health Questionnaire-9; RT = response time; AI = anterior insula; rsFC = resting state functional connectivity; TPJ = temporoparietal junction; ACC = anterior cingulate cortex. ☨ indicates essential predictors exceed the average absolute coefficient of all retained variables (.05).

*B. Cognitive control performance and rsFC in cognitive/emotional control network regions as predictors of symptom change during iCBT (Stage 3), controlling for additional demographic and clinical covariates*

At baseline, the MAC group had higher MASQ anhedonic depression scores, p = 0.048 (Table S4); there were no other significant group differences in MASQ or BAI scores. We entered baseline PHQ-9 scores into the model, along with variables retained at Stage 1 and Stage 2, including age, treatment group, Flanker RT, Gratton accuracy, Post-error accuracy, right AI – TPJ rsFC, and left AI – right AI rsFC. Additional covariates included gender, MASQ Anxious Arousal, MASQ Anhedonic Depression, and Beck Anxiety Inventory scores. Essential variables retained in the final model were identical to the essential predictors in the Stage 3 model presented in the main manuscript and included demographic/treatment variables (treatment group and gender); Flanker RT; Right AI-TPJ rsFC; Right AI-Left AI rsFC; and the interactions between treatment group and: Baseline PHQ-9, age, gender, Flanker RT, Gratton accuracy, post-error accuracy, Right AI-TPJ rsFC, and Right AI-Left AI rsFC. See Supplementary Table S7.

Supplementary Table S7

| **Variable** | **Proportion of replicates with non-zero coefficients** | **Average Coefficient** |
| --- | --- | --- |
| Tx group (MDD)☨ | 1 | -0.14 |
| Baseline PHQ-9 | 1 | 0.051 |
| Age | 0.9999 | 0.041 |
| Gender☨ | 0.9964 | 0.057 |
| MASQ Anhedonic Depression | 0.9963 | 0.03 |
| MASQ Anxious Arousal | 0.9913 | 0.018 |
| Baseline BAI | 0.9497 | 0.012 |
| Flanker RT☨ | 1 | 0.062 |
| Gratton accuracy | 0.9327 | 0.006 |
| Gratton RT | 0.9994 | -0.029 |
| Post-error RT | 0.9999 | -0.037 |
| Right AI-TPJ rsFC☨ | 1 | 0.063 |
| Right AI-Left AI rsFC☨ | 1 | -0.058 |
| Dorsal ACC-TPJ rsFC | 0.9904 | -0.016 |
| Dorsal ACC-left AI rsFC | 0.9863 | 0.023 |
| Tx group (MDD) * Baseline PHQ-9☨ | 0.9997 | 0.061 |
| Tx group (MDD) * Age☨ | 1 | 0.073 |
| Tx group (MDD) * Gender☨ | 0.9999 | -0.12 |
| Tx group (MAC) * Flanker accuracy | 0.9327 | 0.011 |
| Tx group (MDD) * Flanker accuracy | 0.9632 | -0.026 |
| Tx group (MDD)*Flanker RT☨ | 1 | 0.072 |
| Tx group (MDD) * Gratton accuracy☨ | 1 | -0.116 |
| Tx group (MAC) * Post-error accuracy | 0.9999 | -0.041 |
| Tx group (MDD) * Post-error accuracy☨ | 1 | 0.088 |
| Tx group (MDD) * Post-error RT | 0.9327 | 0.008 |
| Tx group (MDD) * Right AI-TPJ rsFC☨ | 1 | 0.109 |
| Tx group (MDD) * Right AI-Left AI rsFC☨ | 1 | -0.082 |
| Tx group (MDD) * Dorsal ACC-TPJ rsFC | 0.9769 | -0.026 |

*Note.* Tx = treatment; MDD = Major Depressive Disorder; PHQ-9 = Patient Health Questionnaire-9; MASQ = Mood and Anxiety Symptom Questionnaire; BAI = Beck Anxiety Inventory; RT = response time; AI = anterior insula; TPJ = temporoparietal junction; rsFC = resting state functional connectivity; ACC = anterior cingulate cortex. ☨ indicates essential predictors exceed the average absolute coefficient of all retained variables (.054).

*C. Elastic net regressions predicting Post-treatment PHQ-9 scores using only participants with complete data (i.e., with no imputation of missing data)*

37 participants had complete data on all variables.

Stage 1: Cognitive control performance as a predictor of symptom change during iCBT.

We entered basic demographic/treatment variables, including age, gender, treatment group, and baseline PHQ-9 score into the model, along with Flanker performance variables including Flanker accuracy, Flanker RT, Gratton accuracy, Gratton RT, post-error accuracy, and post-error RT. Essential variables retained in the final model included demographic/treatment variables (treatment group and gender) and the interactions between treatment group and: Gratton accuracy and post-error accuracy. Given the lower number of participants, we used a threshold of 5000/10000 replicates for variable retention. See Supplementary Table S8.

Stage 2: Resting state functional connectivity in cognitive/emotional control network regions as a predictor of symptom change during iCBT.

We entered basic demographic/treatment variables, including age, gender, treatment group, and baseline PHQ-9 score into the model, along with connectivity values among dACC, left AI, right AI, and TPJ (Figure 1) ROIs. Essential variables retained in the final model included demographic/treatment variables (treatment group and gender). See Supplementary Table S8.

Stage 3: Cognitive control performance and rsFC in cognitive/emotional control network regions as predictors of symptom change during iCBT.

We entered baseline PHQ-9 scores into the model, along with variables retained at Step 1 and Step 2, including treatment group, gender, Baseline PHQ-9, Flanker accuracy, Flanker RT, Gratton RT, Gratton accuracy, Post-error RT, and right AI – TPJ rsFC. Essential variables retained in the final model included demographic/treatment variables and the interactions between treatment group and Gratton accuracy and post-error accuracy. See Supplementary Table S8.

Supplementary Table S8

| **Variable** | **Proportion of replicates with non-zero coefficients** | **Average Coefficient** |
| --- | --- | --- |
| **Stage 1** | |  |
| **Tx group (MDD)☨** | 1 | -0.273 |
| Baseline PHQ-9 | 0.6465 | 0.027 |
| **Gender☨** | 1 | 0.34 |
| Flanker RT | 1 | 0.068 |
| Gratton accuracy | 0.6283 | 0.038 |
| Gratton RT | 0.9948 | -0.07 |
| Post-error RT | 0.7481 | -0.047 |
| Tx group (MDD) * Baseline PHQ-9 | 0.5265 | 0.039 |
| Tx group (MDD) * Age | 0.6647 | 0.045 |
| Tx group (MDD) * Flanker accuracy | 0.6041 | -0.046 |
| Tx group (MDD) * Flanker RT | 0.6887 | 0.048 |
| **Tx group (MDD) * Gratton accuracy☨** | 1 | -0.234 |
| Tx group (MDD) * Gratton RT | 0.6299 | -0.095 |
| **Tx group (MDD) * Post-error accuracy☨** | 1 | 0.183 |
| Tx group (MDD) * Post-error RT | 0.6457 | 0.066 |
| **Stage 2** | |  |
| **Tx group (MDD)☨** | 1 | -0.32 |
| Gender☨ | 1 | 0.272 |
| Right AI-TPJ rsFC | 0.9981 | 0.057 |
| **Stage 3** | |  |
| **Tx group (MDD)☨** | 1 | -0.239 |
| Baseline PHQ-9 | 0.9397 | 0.037 |
| **Gender☨** | 1 | 0.272 |
| Flanker RT | 0.999 | 0.063 |
| Gratton accuracy | 0.6836 | 0.015 |
| Gratton RT | 0.9968 | -0.052 |
| Post-error RT | 0.959 | -0.04 |
| Right AI-TPJ rsFC | 0.9993 | 0.074 |
| Tx group (MDD) * Baseline PHQ-9 | 0.8199 | 0.042 |
| Tx group (MAC) * Age | 0.6751 | -0.008 |
| Tx group (MDD) * Age | 0.9099 | 0.045 |
| Tx group (MAC) * Flanker accuracy | 0.6706 | -0.004 |
| Tx group (MDD) * Flanker accuracy | 0.8525 | -0.043 |
| Tx group (MDD) * Flanker RT | 0.917 | 0.047 |
| **Tx group (MDD) * Gratton accuracy☨** | 0.9987 | -0.199 |
| Tx group (MDD) * Gratton RT | 0.888 | -0.074 |
| Tx group (MAC) * Post-error accuracy | 0.9912 | -0.057 |
| **Tx group (MDD) * Post-error accuracy☨** | 0.9997 | 0.136 |
| Tx group (MDD) * Post-error RT | 0.8698 | 0.052 |

*Note.* Tx = treatment; RT = response time; AI = anterior insula; TPJ = temporoparietal junction; rsFC = resting state functional connectivity.

Variables in ***bold italics*** are significant in models of both imputed and non-imputed data. ☨ indicates essential predictors exceed the average absolute coefficient of all retained variables (Step 1: .104, Step 2: .172, Step 3: .076).

D. *Variables associated with baseline depression*

We applied a three-stage approach similar to that for the prediction of post-treatment PHQ-9 scores. In Stage 1, we entered age, gender, and the Flanker behavioral variables as predictors of baseline PHQ-9 scores. We used a cutoff of 7500 replicates, as in the primary analyses. Essential variables retained in the final model included treatment group, Post-error RT and the interaction between treatment group and Post-error RT. In Stage 2, we entered age, gender, and the rsFC variables as predictors of baseline PHQ-9 scores. No variables (besides the intercept) were retained in the final model. In Stage 3, we entered variables retained at Stage 1, including treatment group, Flanker RT, Gratton RT, post-error accuracy, and post-error RT. Essential variables retained in the final model included treatment group, post-error accuracy, post-error RT, and the interactions between treatment group and: Flanker RT and post-error RT. See Supplementary Table S9.

Supplementary Table S9

| **Variable** | **Proportion of replicates with non-zero coefficients** | **Average Coefficient** |
| --- | --- | --- |
| **Stage 1** | |  |
| Tx group (MDD)☨ | 0.9676 | -0.196 |
| Gratton RT | 0.9603 | -0.045 |
| Post-error accuracy | 0.9613 | 0.154 |
| Post-error RT☨ | 0.9691 | -0.307 |
| Tx group (MDD) * Flanker RT | 0.8675 | -0.083 |
| Tx group (MDD) * Post-error accuracy | 0.9691 | 0.108 |
| Tx group (MDD) * Post-error RT☨ | 0.9566 | 0.309 |
| **Stage 2** | |  |
| N/A |  |  |
| **Stage 3** | |  |
| Tx group (MDD)☨ | 1 | -0.256 |
| Gratton RT | 1 | -0.06 |
| Post-error accuracy☨ | 1 | 0.273 |
| Post-error RT☨ | 1 | -0.509 |
| Tx group (MAC) * Flanker RT☨ | 1 | 0.284 |
| Tx group (MDD) * Flanker RT | 1 | -0.088 |
| Tx group (MDD) * Post-error accuracy | 1 | 0.061 |
| Tx group (MDD) * Post-error RT☨ | 1 | 0.585 |

*Note.* Tx = treatment; RT = response time. ☨ indicates essential predictors exceed the average absolute coefficient of all retained variables (Step 1: .16, Step 2: N/A, Step 3: .248).

*E. Entering all variables simultaneously into the elastic net as a single step, using HAMD scores instead of PHQ-9 scores*

In response to an inquiry regarding our use of PHQ-9 scores instead of HAMD scores, we repeated the main analysis using HAMD scores. We entered all variables into the model in a single stage (as in A above). Given the large number of entered variables, we used a lower cutoff (5000 out of 10,000 replicates) to consider a variable retained. We entered basic demographic/treatment variables, including age, gender, treatment group, and baseline HAMD score into the model, along with Flanker performance variables including Flanker accuracy, Flanker RT, Gratton accuracy, Gratton RT, post-error accuracy, and post-error RT; and connectivity values among dACC, left AI, right AI, and TPJ ROIs (n = 32 variables, including interactions and the intercept).

See Supplement section A above for results obtained using the PHQ-9 and Supplementary Table S10 for results obtained using the HAMD. Treatment group and Flanker RT were retained as essential predictors in the model regardless of whether PHQ-9 or HAMD was used as the measure of depression. Right AI-left AI rsFC, and the interactions between treatment group and: age and right AI-left AI rsFC were retained in both the PHQ-9 and HAMD models but did not exceed the average absolute coefficient of all retained variables in the HAMD model. Findings related to Gratton and post-error accuracy and right AI-TPJ connectivity were specific to the PHQ-9 analysis. This may be related to clinically relevant differences between PHQ-9 (self-report) and HAMD (clinician report), see e.g. ^12,13^. Since use of HAMD is unlikely to persist in clinical practice of iCBT given that it is not self-administered, we opted to focus the bulk of the paper on prediction of reduction in PHQ-9 scores (e.g. ^14^).

Supplementary Table S10

| **Variable** | **Proportion of replicates with non-zero coefficients** | **Average Coefficient** |
| --- | --- | --- |
| Tx group (MDD)☨ | 1 | -0.427 |
| Age | 1 | 0.046 |
| Flanker RT☨ | 1 | 0.228 |
| Right AI-Left AI rsFC | 0.9971 | -0.034 |
| Tx group (MDD) * Age | 0.9694 | 0.016 |
| Tx group (MDD) * Right AI-Left AI rsFC | 1 | -0.162 |

*Note.* Tx = treatment; RT = response time; AI = anterior insula; rsFC = resting state functional connectivity. ☨ indicates essential predictors exceed the average absolute coefficient of all retained variables (.165).

*F. Entering all variables simultaneously into a binomial logistic elastic net as a single step, using response status instead of PHQ-9 scores*

In response to an anonymous reviewer regarding our use of continuous PHQ-9 scores instead of response status, we repeated the analysis using response status defined as a ≥50% decrease in PHQ-9 scores. We entered all variables into the model in a single stage (as in A above). Given the large number of variables, we used a lower cutoff (5000 out of 10,000 replicates) to consider a variable retained. We entered basic demographic/treatment variables, including age, gender, treatment group, and baseline PHQ-9 score, into the model, along with Flanker performance variables including Flanker accuracy, Flanker RT, Gratton accuracy, Gratton RT, post-error accuracy, and post-error RT; and connectivity values among dACC, left AI, right AI, and TPJ ROIs (n = 32 variables, including interactions and the intercept).

See Supplement section A above for results obtained using the continuous PHQ-9 scores and Supplementary Table S11 for results obtained using response status.

When comparing the single step model predicting response status with the stage 3 model predicting continuous post-treatment PHQ-9 scores, the interactions between treatment group and: Flanker RT and Gratton accuracy were retained as essential predictors in the model regardless of whether continuous post-treatment PHQ-9 scores or response status were used as the measure of treatment outcome. Flanker RT, Right AI-TPJ rsFC, Right AI-left AI rsFC, and the interactions between treatment group and age were retained in both the continuous post-treatment PHQ-9 scores and response status models but did not exceed the average absolute coefficient of all retained variables in the response status model. Although the interaction between treatment group and baseline PHQ-9 scores were retained in both models, the direction of associations was opposite. Findings related to the left AI-TPJ and the interaction between treatment group and dACC-right AI rsFC were specific to the response status model. The interactions between treatment group and: post-error accuracy, right AI-TPJ rsFC and right AI-left AI rsFC were retained as essential predictors in Stage 3 of the model predicting continuous post-treatment PHQ-9 scores. All variables in the response status model were retained in the single step model predicting continuous post-treatment PHQ-9 scores (the interaction between treatment group and baseline PHQ-9 and dACC-right AI rsFC showed opposite patterns in both models, however). Overall, the model predicting response status showed similar results to the model predicting continuous PHQ-9 scores. Differences in findings may be due to lower variance in the binary response status variable compared to the continuous PHQ-9 score.

Supplementary Table S11

| **Variable** | **Proportion of replicates with non-zero coefficients** | **Average Coefficient** |
| --- | --- | --- |
| **Flanker RT** | **1** | **-0.317** |
| Left AI-TPJ rsFC | 0.9965 | 0.192 |
| **Right AI-TPJ rsFC** | **1** | **-0.424** |
| **Right AI-Left AI rsFC☨** | **1** | **0.503** |
| **Tx group (MDD) * Baseline PHQ-9^+^** | **0.9713** | **0.121** |
| **Tx group (MDD) * Age** | **1** | **-0.122** |
| **Tx group (MDD) * Flanker RT☨** | **1** | **-0.917** |
| **Tx group (MDD) * Gratton accuracy☨** | **1** | **1.09** |
| Tx group (MDD) * dACC-right AI rsFC | 0.6271 | -0.062 |

*Note.* Tx = treatment; RT = response time; AI = anterior insula; rsFC = resting state functional connectivity. ☨ indicates essential predictors exceed the average absolute coefficient of all retained variables (.449). **^+^** indicates predictors that show the opposite direction compared to the model predicting continuous PHQ-9 scores

Variables in ***bold italics*** are significant in the Stage 3 model with continuous post-treatment PHQ-9 scores and the one-stage response status.

References

1. Kroenke, K., Spitzer, R. L. & Williams, J. B. W. The PHQ-9. *J. Gen. Intern. Med.* **16**, 606–613 (2001).

2. Clark, L. A. & Watson, D. Tripartite Model of Anxiety and Depression: Psychometric Evidence and Taxonomic Implications. 21 (1991).

3. Watson, D. *et al.* Testing a tripartite model: I. Evaluating the convergent and discriminant validity of anxiety and depression symptom scales. *J. Abnorm. Psychol.* **104**, 3–14 (1995).

4. Watson, D. & Walker, L. M. The long-term stability and predictive validity of trait measures of affect. *J. Pers. Soc. Psychol.* **70**, 567–577 (1996).

5. Beck, A. T., Epstein, N., Brown, G. & Steer, R. A. An inventory for measuring clinical anxiety: psychometric properties. *J. Consult. Clin. Psychol.* **56**, 893 (1988).

6. Beck, A. T., Epstein, N., Brown, G. & Steer, R. Beck anxiety inventory. *J. Consult. Clin. Psychol.* (1993).

7. Beck, A. T. & Steer, R. A. Manual for the Beck anxiety inventory. *San Antonio TX Psychol. Corp.* (1990).

8. Osman, A., Kopper, B. A., Barrios, F., Gutierrez, P. M. & Bagge, C. L. Reliability and Validity of the Beck Depression Inventory--II With Adolescent Psychiatric Inpatients. *Psychol. Assess.* **16**, 120–132 (2004).

9. Olvet, D. M. & Hajcak, G. The stability of error‐related brain activity with increasing trials. *Psychophysiology* **46**, 957–961 (2009).

10. Whitfield-Gabrieli, S. & Nieto-Castanon, A. Conn : A Functional Connectivity Toolbox for Correlated and Anticorrelated Brain Networks. *Brain Connect.* **2**, 125–41 (2012).

11. Behzadi, Y., Restom, K., Liau, J. & Liu, T. T. A component based noise correction method (CompCor) for BOLD and perfusion based fMRI. *NeuroImage* **37**, 90–101 (2007).

12. Cuijpers, P., Li, J., Hofmann, S. G. & Andersson, G. Self-reported versus clinician-rated symptoms of depression as outcome measures in psychotherapy research on depression: A meta-analysis. *Clin. Psychol. Rev.* **30**, 768–778 (2010).

13. Ma, S. *et al.* The Patient Health Questionnaire-9 vs. the Hamilton Rating Scale for Depression in Assessing Major Depressive Disorder. *Front. Psychiatry* **12**, 747139 (2021).

14. Karyotaki, E. *et al.* Internet-based cognitive behavioral therapy for depression: a systematic review and individual patient data network meta-analysis. *JAMA Psychiatry* **78**, 361–371 (2021).
